# Supplementary material for: The interactome of intact mitochondria by cross-linking mass spectrometry provides evidence for coexisting respiratory supercomplexes
Source: Mol Cell Proteomics. 2017 Dec 8;17(2):216–32. doi: 10.1074/mcp.RA117.000470 (PMC5795388; doi:10.1074/mcp.RA117.000470)
Supplement: Supplemental Data [file supp_RA117.000470_133922_0_supp_23978_fzffwf.zip › spectra_annotation/mito_spectra_annotation/449-1-10-1-10-1.pdf]

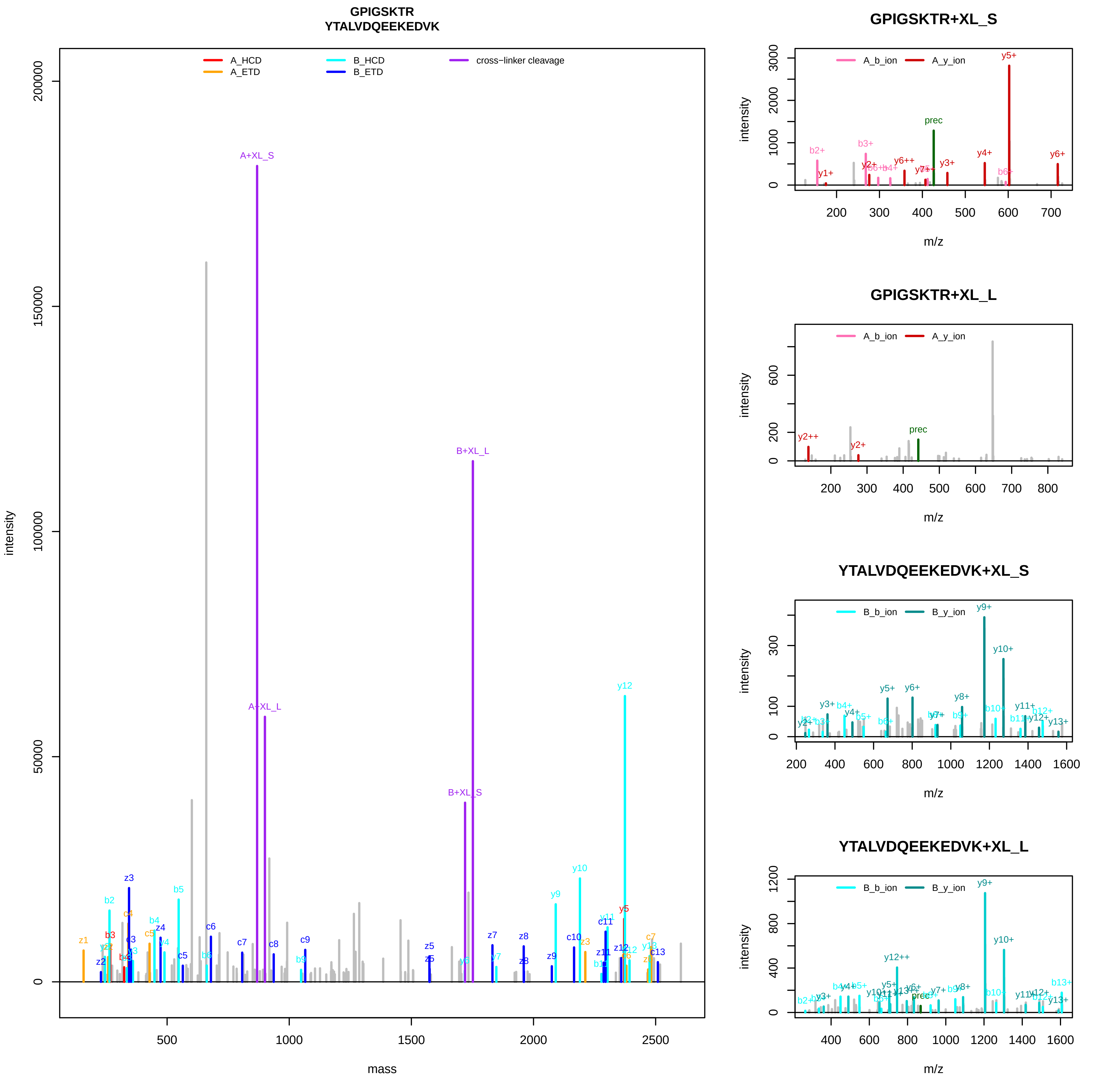
GPIGSKTR
YTALVDQEEKEDVK
A_HCD
A_ETD
B_HCD
B_ETD
cross−linker cleavage
A+XL_S
A+XL_L
B+XL_L
B+XL_S
y12
y10
y9
b5
b2
z3
c6
c7
c8
c9
z7
z8
z9
c10
c11
y5
intensity
mass
GPIGSKTR+XL_S
A_b_ion
A_y_ion
y5+
prec
y6+
y4+
y3+
b3+
b2+
m/z
GPIGSKTR+XL_L
y2++
y2+
YTALVDQEEKEDVK+XL_S
B_b_ion
B_y_ion
y9+
y10+
y8+
y6+
y5+
YTALVDQEEKEDVK+XL_L
y12++
y13+
